# TSPAN1-elevated FAM110A promotes pancreatic cancer progression by transcriptionally regulating HIST1H2BK

**DOI:** 10.7150/jca.66404

**Published:** 2022-01-01

**Authors:** Hua Huang, Huan Li, Ting Zhao, Aamir Ali Khan, Ruining Pan, Sijia Wang, Shensen Wang, Xinhui Liu

**Affiliations:** 1 Center of Excellence for Environmental Safety and Biological Effects, Beijing International Science and Technology Cooperation Base for Antiviral Drugs, Faculty of Environment and Life, Beijing University of Technology, Beijing 100124, China.; 2 Department of Nutrition, The Affiliated Hospital of Qingdao University, Qingdao 266000, China.

**Keywords:** Pancreatic cancer, FAM110A, HIST1H2BK, TSPAN1, G9a

## Abstract

**Background:** FAM110A belongs to the FAM110 family, which mainly functions in biological processes associated with the cell cycle. However, the biological functions in which FAM110A participates are largely undefined. In particular, its potential role in cancer remains unknown. The goal of this study was to uncover the role and mechanism of FAM110A in pancreatic cancer.

**Methods:** Based on bioinformatics databases, qPCR and Western blot assays, we verified the elevated expression level of FAM110A in PDAC. Subsequently, FAM110A, HIST1H2BK and TSPAN1 overexpression or knockdown stable transfected cells were employed for biological functions' studies to explore the role in PDAC *in vitro* and *in vivo*. RNA-Seq, Western blot and luciferase-reporter assays were used to explore mechanism of FAM110A action in PDAC, and the involved pathway was verified by tumor phenotypic rescue experiments.

**Results:** In this study, we demonstrated for the first time that FAM110A is an oncogene that promotes cell proliferation, migration, invasion and tumorigenesis in pancreatic cancer. HIST1H2BK was identified as the downstream target of FAM110A, while the promotion effect caused by FAM110A overexpression could be abolished by HIST1H2BK knockdown. Moreover, for the first time, we revealed the oncogenic role of HIST1H2BK in pancreatic cancer, and the tumor-promoting capacity of HIST1H2BK may be associated with its regulatory effect on G9a. In addition, we demonstrated that TSPAN1 displayed a positive transcriptional regulatory effect on FAM110A.

**Conclusions:** Collectively, FAM110A plays an oncogenic role in PDAC, and the newly identified TSPAN1/FAM110A/HIST1H2BK/G9a pathway is involved in the modulation of pancreatic cancer progression and provides a novel prognostic and therapeutic strategy for pancreatic cancer treatment.

## Introduction

Pancreatic ductal adenocarcinoma (PDAC) is recognized as the most aggressive type of cancer, with the lowest 5‐year relative survival rate (9%), and is the leading cause of cancer death among all cancers 1. The extremely poor prognosis of PDAC is due to its difficulty in early detection and rapid metastasis 2. As a result, the majority of pancreatic cancer patients are diagnosed at an advanced stage of cancer. Therefore, identifying novel biomarkers and elucidating their molecular mechanisms in PDAC are urgently required for its effective treatment.

FAM110A belongs to the FAM110 (family with sequence similarity 110) family, consisting of three members (A, B and C). FAM110 proteins are localized in centrosomes and accumulate at the microtubule organization center and spindle poles in interphase and mitosis 3. FAM110 proteins mainly function in biological processes associated with the cell cycle, such as cell proliferation and differentiation 4. FAM110B has been indicated as a tumor differentiation-related gene that decreases along with the tumor differentiation grades in pancreatic adenocarcinoma. The potential oncogenic role of FAM110B has been demonstrated in castration-resistant prostate cancer 5. FAM110C has been suggested to affect microtubule cytoskeleton organization 3 and to play roles in cell adhesion and migration 6. A very recent paper reported that FAM110A localized to the proximal spindle and spindle poles during mitosis, and thus is needed for mitotic progression 7. However, the biological functions in which FAM110A participates are largely undefined. In particular, a role in cancer progression has never been reported. In this study, we first suggested the critical oncogenic role of FAM110A and investigated its underlying mechanisms in PDAC.

Tetraspanin 1 (TSPAN1) is a member of the tetraspanin family, which is a family of small transmembrane proteins. TSPAN1 has been reported to be elevated in many human cancers and to be involved in tumor progression 8-12. Our previous study has revealed that TSPAN1 is an oncogene that promotes cell proliferation, migration, invasion and tumorigenesis in PDAC 8. In addition, RNA-Seq with TSPAN1-modulated cells (TSPAN1-overexpressing and knockdown) identified FAM110A presented identical trends as TSPAN1 in PANC-1 cells. Thus, TSPAN1 displayed the potent regulatory effect on FAM110A.

Histone cluster 1 H2B family member k (HIST1H2BK), also known as H2BC12, is a subtype of histone H2B. Mutations in the core histone result in nucleosome dysfunction and abnormal gene expression, which may lead to an oncogenic program. A mutation in histone H2B, as a new mechanism of epigenetic dysfunction, occurs in diverse cancers and represents a new class of oncogenic drivers 13, 14. A recent study demonstrated the increased levels of several subtypes of histone H2B after BaP-induced malignant cell transformation, and PARG silencing could inhibit BaP-induced carcinogenesis by downregulating the expression of H2B, indicating the important role of H2B in oncogenesis 15. Previous bioinformatic studies showed the high expression level of HIST1H2BK in various types of cancer, which was associated with poor prognosis 16-19. However, little is known about the biological functions of HIST1H2BK, especially its role in cancer.

G9a, a histone methyltransferase, catalyzes the methylation of H3K9 (histone 3 lysine 9) and H3K27 (histone 3 lysine 27) 20. G9a participates in diverse biological processes, including DNA replication and DNA damage and repair, and catalyzes methylation processes such as methylation of p53, DNMT1, HDAC1 and KLF12 21-24. Additionally, G9a has been shown to be overexpressed in multiple human cancers and involved in tumor development 25-28. Interestingly, HIST1H2BK was found in the histone microclusters on chromosome 6p21.33, where G9a is one of the genes localized in the region, indicating a possible correlation.

In this study, we investigated the potential oncogenic activity and the underlying mechanisms of FAM110A in PDAC. For the first time, we demonstrated the oncogenic role of FAM110A and HIST1H2BK in PDAC. We proposed that a newly identified TSPAN1/FAM110A/HIST1H2BK/G9a axis might enrich the current understanding of PDAC progression and provide a novel therapeutic target.

## Materials and Methods

### Cell culture

Human pancreatic cancer cell lines (PANC-1, BXPC3, ASPC1) and the human pancreatic ductal epithelial cell line (HPDE6-C7) were obtained from the Cell Bank of the Chinese Academy of Sciences. The cells were cultured in DMEM high-glucose medium (Biological Industries: 01-052-1A) containing 10% FBS (Biological Industries: 04-007-1A), 100 units/mL penicillin and 100 μg/mL streptomycin (HyClone, Logan, UT, USA). Cells were incubated at 37 °C in a humidified atmosphere of 5% CO_2_.

### Tissue specimens

Twelve pairs of pancreatic adenocarcinomas and matched adjacent non-tumor tissue specimens were obtained from patients with PDAC who underwent surgical resection at the Affiliated Hospital of Qingdao University. All cancers were verified as pancreatic adenocarcinomas. The specimens were used with informed consent from patients and approval by Ethics Committee of the Affiliated Hospital of Qingdao University (NO. QYFYWILL36631). No patients received preoperative chemotherapy or radiotherapy. The surgical specimen sample information is summarized in Supplementary Table S3.

### Generation of stable cell lines

To generate the FAM110A-OE, HIST1H2BK-OE, TSPAN1-OE, shFAM110A, shTSPAN1 and FAM110A-OE coupled with HIST1H2BK-KD stably transfected cell lines, we subcloned the CDSs of FAM110A (transcript variant 1, NM_031424), HIST1H2BK and TSPAN1 into the pCDH-EF1α-MCS-T2A-Puro lentivirus vector (System Biosciences: CD527A-1) and cloned the shRNA sequences of FAM110A and HIST1H2BK into the pLVX-shRNA2-BSD lentivirus vector upgraded by pLVX-shRNA2 (Clonetech:632179). All plasmids were first purified and packaged into lentivirus and then transfected into PANC-1 cells or BXPC-3 cells. After 36 h of transfection, (5 μg/ml) blasticidin S or (2 μg/ml) puromycin was added to the cultured cells to screen the knockdown and overexpressed cells, respectively. After two weeks of screening with puromycin and blasticidin S, all viable cells were considered positive and were further validated by Western blot and qPCR.

### Quantitative real time-PCR (qPCR)

Total RNA was extracted from cells using TRIzol (Invitrogen, Carlsbad, CA, USA) according to the manufacturer's instructions. Reverse transcription was performed using the HiScript III 1st Strand cDNA Synthesis Kit (Vazyme, Nanjing, China). Gene expression was evaluated via qPCR using ChamQ SYBR Color qPCR Master Mix (Vazyme, Nanjing, China) on an ABI StepOne system (Applied Biosystems, Foster City, CA, USA). Gene expression was evaluated and normalized to the internal control of β-Actin. Primer sequences are shown in Supplementary Table S1.

### Western blotting

Proteins were extracted using cell lysis buffer (Beyotime, Shanghai, China) and a protease and phosphatase inhibitor cocktail (Beyotime, Shanghai, China), separated by 4-20% SDS-PAGE gels and were then subjected to immunoblotting with appropriate primary and secondary antibodies (Supplementary Table S2). Luminescence was visualized using a BeyoECL Moon super sensitivity detection kit (Beyotime, Shanghai, China).

### Promoter Luciferase reporter assay

A promoter sequence 2057 bp in length, ranging from the -2000 base site to the +57 base site of the HIST1H2BK gene, was copied into the modified pGL4.11[luc2P] (Promega, Madison, WI) vector. A promoter sequence 2001 bp in length, ranging from the -2000 base site to the +1 base site of the FAM110A gene, was copied into the modified pGL4.11 (Promega, Madison, WI) vector. The Renilla luciferase gene sequence was added to the vector for the normalization of transfection efficiency. Cells were cotransfected with the pcDNA3.1-FAM110A plasmid or pcDNA3.1 empty vector and 50 ng of the pGL-HIST1H2BK-promoter vector. Firefly luciferase activities and Renilla signals were measured 48 h after transfection using a dual-luciferase reporter assay kit (Promega, Madison, USA) according to the manufacturer's instructions.

### Cell proliferation assay

The cell proliferative potential was analyzed by CCK-8 assays. Cells were seeded in 96-well plates at a cell density of 2×10^3^ cells per well, and 10 µL of CCK-8 (Cell Counting Kit-8, Solarbio, Beijing, China) reagent was added to each well. The plates were incubated at 37 °C for an extra 2 h, and the signal was detected at 450 nm using a microplate reader (Bio-Rad).

### Colony formation assay

Transduced cells were seeded into 6-well plates (300 cells/well) and incubated in a humidified incubator containing 5% CO_2_ at 37 °C for 10-14 days. Cells were washed with PBS two times, fixed with 4% paraformaldehyde and stained with 0.5% crystal violet. After staining, photographs were taken, and the number of purple colonies was counted.

### Wound healing assay

A total of 2.5×10^5^ cells/well were seeded and cultured in 12-well plates to create a confluent monolayer. Horizontal and vertical wounds were scraped after the cells had attached. After 48 h, images of the “scratch closure” were photographed with a microscope, and the remaining uncovered area was measured and matched with the initial scratched area.

### Transwell invasion assay

Cells were harvested and suspended in serum-free DMEM for the cell invasion assay.

A total of 1×10^4^ cells were seeded in the Matrigel-coated upper chamber (8 μm Transwell inserts, BD Biosciences, San Jose, CA), and DMEM with 10% FBS was added to the bottom chamber as an attractant. The plate was incubated for 24 h, and cells on the bottom of the upper chamber were washed with PBS, fixed in 4% paraformaldehyde and stained with 0.5% crystal violet. Stained cells were visualized and counted in three different fields under a light microscope (Leica, USA).

### *In vivo* tumorigenicity assays

The research was approved by the Ethics Committee of Beijing University of technology (HS202105004). The study was performed in accordance with institution guidelines. Female Balb/c nude mice (6-8 weeks of age) were obtained from Weitonglihua Company (Beijing, China) and were housed under a 12 h dark, and 12 h light conditions and a standard temperature of 18-23 °C with humidity of 40-60%. The FAM110A-OE or shFAM110A and corresponding control cells (2×10^6^) were injected subcutaneously into the right and left flanks of nude mice. All the mice were sacrificed using excess carbon dioxide (25% chamber volume per minute) approximately 6 weeks after inoculation. Tumors were isolated, weighed and calculated at the experimental end point. Animal experimenters blindly test numbered cell samples without knowing the sample information.

### Statistical analysis

The data are presented as the mean ± SD from at least three independent experiments. Quantitative results were analyzed using Prism 5.0 software (GraphPad Software, Inc., La Jolla, CA, USA). A two‑tailed Student's paired t‑test was used to test for significance between two groups. Multiple groups were compared by one-way ANOVA. Statistical significance was regarded as * P < 0.05, ** P < 0.01 and ***P < 0.001.

## Results

### Highly expressed FAM110A in human pancreatic cancer

**Figure 1:**
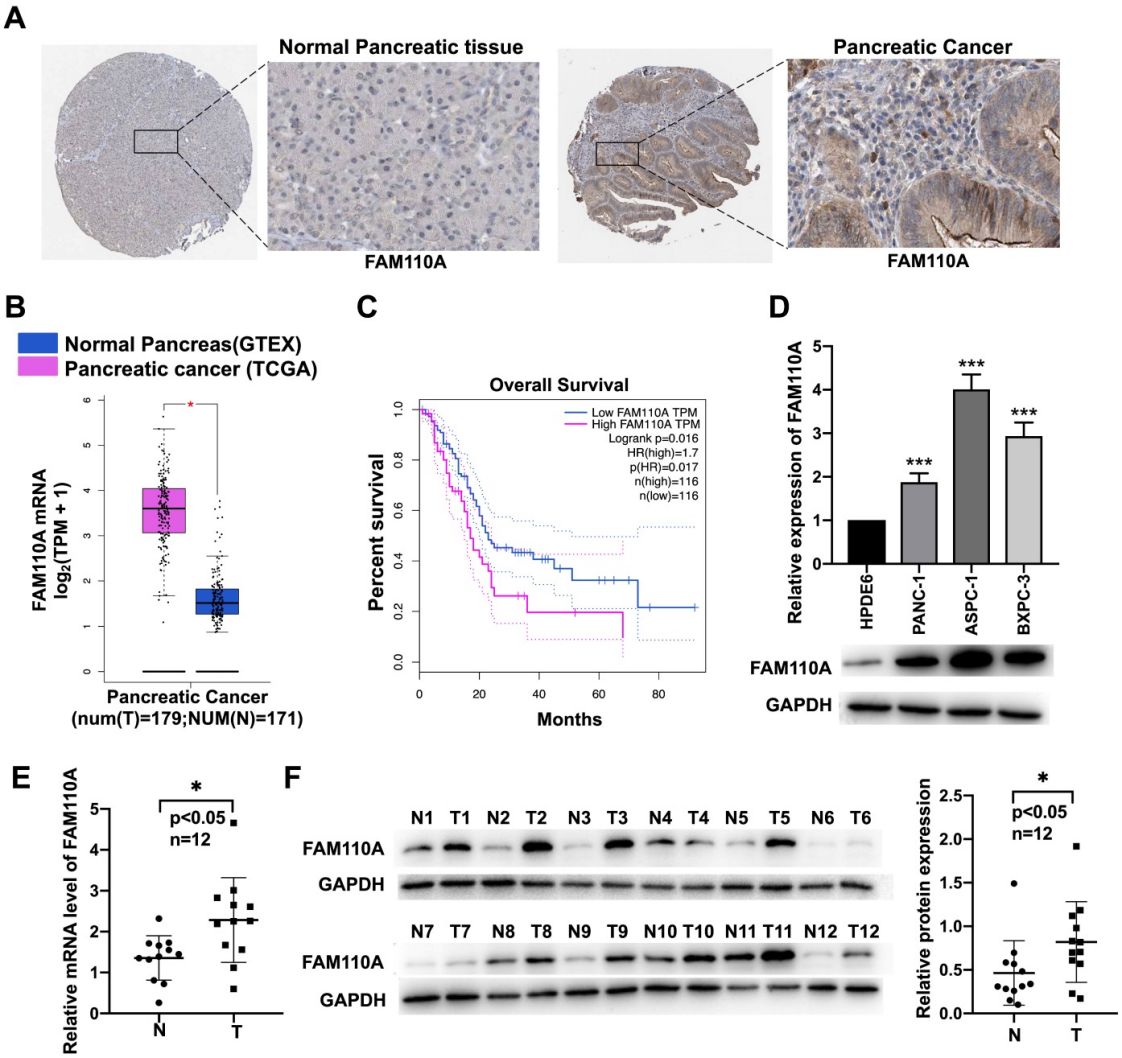
** Upregulated expression of FAM110A in pancreatic cancer cell lines. A,** Representative images of IHC staining for FAM110A protein in normal pancreatic tissue (staining: not detected; intensity: negative) and pancreatic cancer tissue (staining: medium; intensity: moderate) based on the Human Protein Atlas. **B,** Expression of FAM110A in pancreatic cancer according to the TCGA database. **C,** The correlations between FAM110A expression and overall survival in pancreatic cancer analyzed by TCGA database. **D,** The mRNA and protein levels of FAM110A were assessed by qPCR and Western blot assay. **E.** FAM110A mRNA level in 12 pairs of pancreatic cancer tissues and adjacent normal tissues were determined by qPCR. **F**. FAM110A protein expression in 12 pairs of pancreatic cancer tissues and adjacent normal tissues were determined by Western blotting. FAM110A bands were quantitated using ImageJ software, then normalized to GAPDH. Data are presented as the means ± SD (n=3). *P < 0.05; ** P < 0.01 and, *** P < 0.001.

To identify the potential association of FAM110A with cancer, we investigated the expression levels of FAM110A in different cancer tissues and their corresponding adjacent nontumor tissues according to available databases. Due to relatively greater differences in FAM110A expression between pancreatic cancer tumor and nontumor tissues than in other types of cancer, we focused on pancreatic cancer in this study. Data analysis results from the Human Protein Atlas (http://www.proteinatlas.org/) showed signs of upregulation of FAM110A protein in pancreatic cancer compared with normal pancreas. No staining was observed for FAM110A in normal pancreatic tissues, whereas moderate FAM110A staining was observed in ductal-like epithelial cells and surrounding mesenchymal-like cells in pancreatic cancer tissues (Fig. 1A). There was also a significant increase in FAM110A mRNA in pancreatic adenocarcinoma (PAAD) tissues (n=179) compared with adjacent nontumor tissues (n=171) according to the TCGA database (Fig. 1B). The elevated expression of FAM110A in PDAC was confirmed by Quantitative PCR and Western blot of 12 pairs of clinical pancreatic cancer tissues and adjacent normal tissues (Fig.1E, 1F). In addition, pancreatic cancer patients with high FAM110A expression showed significantly reduced overall survival (Fig. 1C, S3). Quantitative PCR and Western blot of three pancreatic cancer cell lines (PANC-1, BXPC3 and ASPC1) and one pancreatic ductal epithelial cell line (HPDE6-C7) confirmed the elevated FAM110A expression levels in PDAC cells (Fig. 1D). These results indicate that FAM110A is upregulated in pancreatic cancer and is associated with poor prognosis.

### FAM110A functions as an oncogene in pancreatic cancer

**Figure 2:**
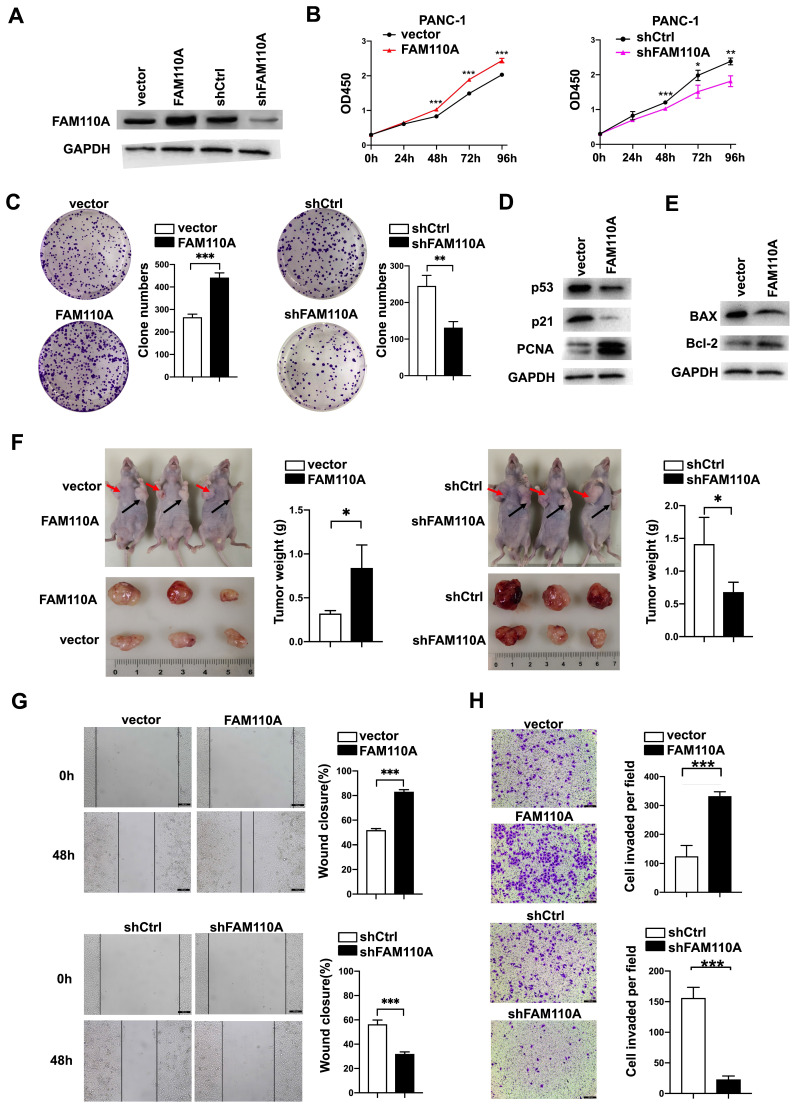
** FAM110A enhances the growth, migration and invasion of pancreatic cancer. A,** Protein level of FAM110A in PANC-1 cells expressing vector, exogenous FAM110A, shCtrl or shFAM110A. **B,** Proliferation of PANC-1 cells expressing exogenous FAM110A, shFAM110A, vector and shCtrl. **C,** Representative images of the clone-forming assay. **D**, Protein level of proliferation-related proteins in PANC-1 cells expressing vector and exogenous FAM110A. **E**, Protein level of apoptosis-related markers in PANC-1 cells expressing vector and exogenous FAM110A. **F,** Images and weight analysis of subcutaneous tumors from the indicated groups(n=3). **G** and **H.** (**G**) Wound healing assays and (**H**) Transwell assays were used to evaluate the migration and invasive potential of FAMM110A overexpression, knockdown and corresponding control cells (scale bar: 100 μm). Data are presented as the means ± SD (n=3). *P < 0.05, **P < 0.01, *** P < 0.001.

To evaluate the biological role of FAM110A in PDAC, we established FAM110A overexpression and knockdown stably transfected cell lines based on PANC-1 and BXPC-3 cells and confirmed the relative expression of FAM110A at the protein level (Fig. 2A). Overexpressed FAM110A significantly increased the proliferation and colony formation capacity, whereas FAM110A knockdown suppressed the proliferation and colony formation capacity (Fig. 2B, C, S1A). The enhanced cell proliferation by FAM110A was confirmed by the upregulation of proliferation-related proteins (Fig. 2D). In addition, we also detected that apoptosis was inhibited in FAM110A overexpressed cells by analyzed the expression of cell apoptosis-related marker Bax and Bcl-2 (Fig. 2E). Furthermore, elevated FAM110A expression enhanced tumor growth in subcutaneous transplantation models in nude mice. Conversely, the tumor volumes and weights in the PANC-1-shFAM110A group were significantly decreased compared with those in the control group (Fig. 2F). These observations suggest that FAM110A promotes the growth of PDAC *in vitro* and *in vivo*.

Wound healing and Transwell assays were performed to study the roles of FAM110A in cell migration and invasion. Our results showed that ectopic expression of FAM110A significantly increased the migration distance of PANC-1 cells, whereas suppression of FAM110A exerted the opposite effect (Fig. 2G). A similar trend was observed: overexpression of FAM110A dramatically increased the number of invasive cells and vice versa (Fig. 2H, S1B). Therefore, FAM110A enhanced the migratory and invasive capabilities of PDAC cells. Collectively, these results indicate that FAM110A plays an important role as a promotor of PDAC progression and may serve as a prognostic marker and therapeutic target for PDAC.

### HIST1H2BK is a downstream target of FAM110A in pancreatic cancer

**Figure 3:**
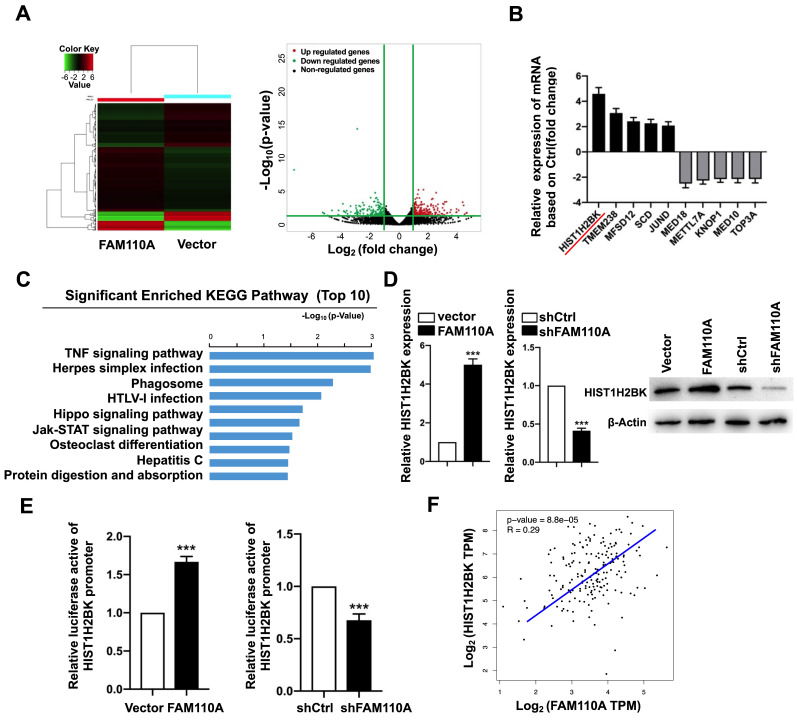
** HIST1H2BK is a downstream target of FAM110A. A,** RNA-Seq identified differentially expressed genes in FAM110A stably overexpressing cells when compared with their corresponding controls. (fold change >2 or 2.0, P < 0.05) **B,** The relative mRNA levels of HIST1H2BK, TMEM238, MFSD12, SCD, JUND, MED18, METTL7A, KNOP1, MED10, and TOP3A in RNA-seq of FAM110A-overexpressing PANC-1 cells. **C,** KEGG pathway enrichment analysis of DEGs in PANC-1 cells upon FAM110A overexpression was performed. The top 10 enriched signaling pathways are shown and are ranked on the basis of log10(p-value).** D,** The mRNA and protein levels of HIST1H2BK in FAM110A-overexpressing and FAM110A-knockdown PANC-1 cells were detected using qRT-PCR and Western blot, respectively. **E,** A luciferase reporter assay evaluated the effect of FAM110A overexpression on the transcriptional activity of HIST1H2BK in PANC-1 cells. Firefly luciferase activity was normalized to Renilla luciferase activity and expressed as the mean ± SD. **F,** The correlation between FAM110A and HIST1H2BK in PDAC based on the TCGA dataset. Data are shown as the mean ± S.D. (n = 3) *P < 0.05, **P < 0.01 and *** P < 0.001.

To explore oncogenic mechanism of FAM110A in PDAC, we examined the subcellular localization of FAM110A. We observed that FAM110A localized to the nucleus and centrosomes, where some of the cells showed nucleolus enrichment (Fig. S4). We assume that FAM110A may be involved in transcriptional regulation. Therefore, we performed RNA-Seq using FAM110A-overexpressing and control cells. We observed that 259 genes were upregulated and 184 genes were downregulated after FAM110A overexpression (fold change≥2, P < 0.05) (Fig. 3A). KEGG pathway enrichment analysis revealed that the differentially expressed genes (DEGs) were significantly enriched in pathways associated with tumorigenesis, including the TNF signaling pathway, the Hippo signaling pathway, and the JAK-STAT signaling pathway (Fig. 3C). Among the DEGs, we identified the top five genes with elevated expression levels and the top five genes whose levels were downregulated in FAM110A-overexpressing cells (Fig. 3B). HIST1H2BK is the most strongly upregulated gene in response to FAM110A overexpression, whose biological functions are largely unknown. This aroused our great interest. qPCR and Western blot assays confirmed the elevated level of HIST1H2BK when FAM110A was upregulated and vice versa (Fig. 3D). In addition, a luciferase promoter reporter assay was performed to evaluate the transcriptional regulatory role of FAM110A on HIST1H2BK. FAM110A overexpression increased the activity of the HIST1H2BK promoter, whereas FAM110A knockdown had the opposite effect (Fig. 3E). Our results are consistent with the correlation analysis based on the TCGA dataset. The expression of HIST1H2BK presented a positive correlation with FAM110A mRNA levels in PDAC tissues (Fig. 3F). These results demonstrate that HIST1H2BK is a direct target of FAM110A in PDAC.

### FAM110A promotes tumorigenesis in PDAC by regulating HIST1H2BK

**Figure 4:**
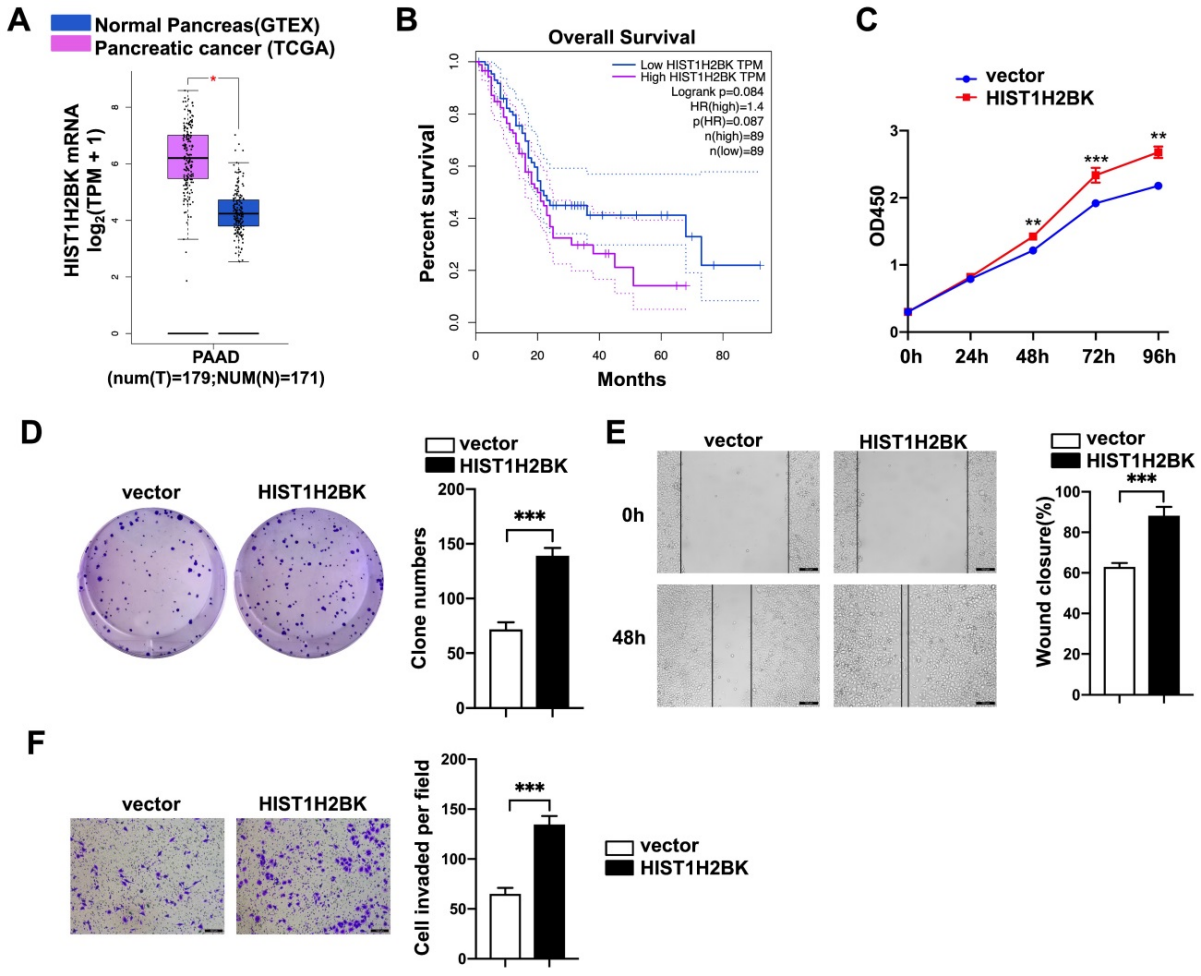
** HIST1H2BK promotes pancreatic cancer progression. A,** Expression of HIST1H2BK in pancreatic cancer according to the TCGA database. **B,** Correlations between the expression of HIST1H2BK and patient survival rate (n = 89). **C** and** D,** Cell proliferation rate (**C**) and colony-forming capacity (**D**) of FAM110A-overexpressing cells and control cells. **E** and **F,** Wound healing assays (**E**) and Transwell assays (**F**) were used to evaluate the involvement of HIST1H2BK in the migration and invasion of PANC-1 cells (scale bars=100 μm). Data are shown as the mean ± SD. n = 3 *P < 0.05, **P < 0.01 and *** P < 0.001 were considered significant.

**Figure 5:**
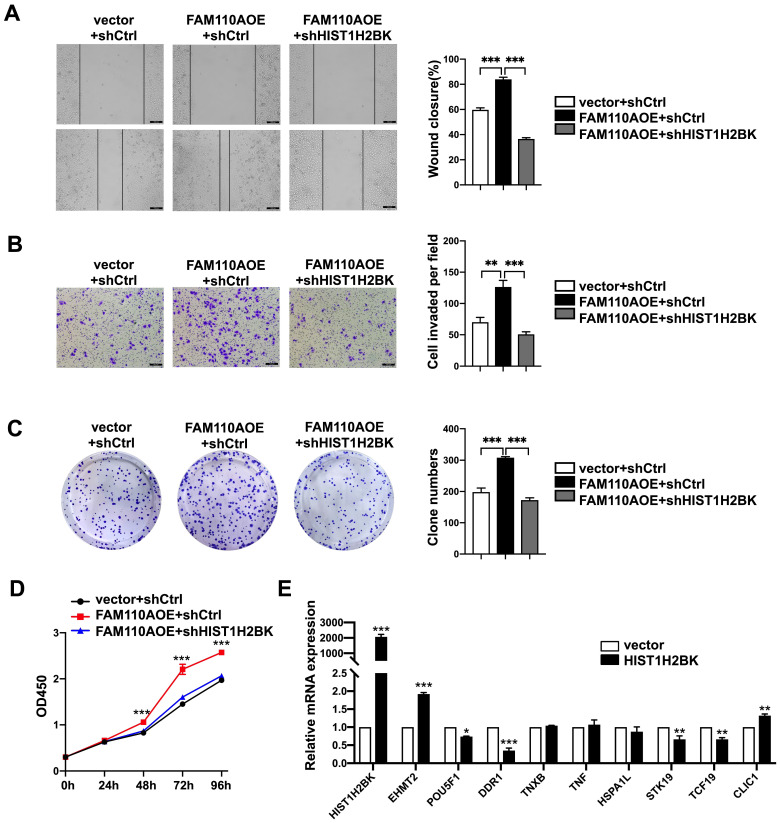
** FAM110A promotes tumorigenesis in PDAC by regulating HIST1H2BK. A, B, C and D,** HIST1H2BK knockdown abolished the promotion of cell proliferation (**D**), colony formation capacity (**C**), migration (**A**) and invasion (**B**) caused by overexpression of FAM110A in PANC-1 cells. **E,** The mRNA levels of EHMT2, POU5F1, DDR1, TNXB, TNF, HSPA1L, STK19, TCF19, and CLIC1 in HIST1H2BK-overexpressing PANC-1 cells using qPCR. Data are shown as the mean ± SD. n = 3 *P < 0.05, **P < 0.01 and *** P < 0.001 were considered significant.

According to limited bioinformatic studies, HIST1H2BK has been shown to be upregulated in breast, pancreatic, and lung cancers and glioma 16, 18, 19. However, there is no direct evidence for the role of HIST1H2BK in cancer. According to the TCGA database, the expression level of HIST1H2BK was upregulated in human pancreatic cancer specimens (Fig. 4A) and was associated with a poor survival rate (Fig. 4B). To validate the role of HIST1H2BK in PDAC, we stably overexpressed HIST1H2BK in PANC-1 cells. HIST1H2BK overexpression significantly promoted cell proliferation (Fig. 4C) and colony formation (Fig. 4D) compared with the control cells. The HIST1H2BK-overexpressing cells also showed increased migration and invasive abilities (Fig. 4E, F). The oncogenic role of HIST1H2BK in PDAC was confirmed in BXPC-3 cells that shHIST1H2BK inhibited the cell proliferation (Fig. S2A) and invasion (Fig. S2B) compared with the control cells. To further determine the functional association between FAM110A and HIST1H2BK, we stably knocked down HIST1H2BK in FAM110A-overexpressing PANC-1 cells. We found that the tumor-promoting capacity of FAM110A could be markedly abolished by HIST1H2BK knockdown in PDAC (Fig. 5A, B, C, D). In summary, these results reveal that the oncogenic function of FAM110A in PDAC is closely related to its regulatory effect on HIST1H2BK, which also plays a carcinogenic role in PDAC.

### HIST1H2BK promotes tumorigenesis in PDAC by modulating G9a

HIST1H2BK was found in the histone microclusters on chromosome 6p21.33. This area contains the major histocompatibility complex (MHC) class III region, which is a subclass of the MHC region. MHC is an ~4 Mbp region of the genome located on chromosome (Chr) 6. It contains genes encoding highly polymorphic class I and class II histocompatibility proteins, which play important immunologic functions through synergistic effects with T cells. Class I and class II genes are separated by a gene-dense segment of DNA, which is termed the class III region. MHC Class III areas have positioned at least 36 genes, including genes associated with the immune system (C4B, C4A, C2, Bf), tumor necrosis factor (TNFA, TNFB) and heat shock protein 70 (HSP70). Thus, we hypothesized that HIST1H2BK may regulate the expression of the genes in this area by regulating chromatin conformation or influencing DNA densification in this region.

To verify this hypothesis, we selected some tumor-associated genes as candidates from this region and evaluated their expression in HIST1H2BK-overexpressing cells. EHMT2, also known as G9a, was significantly upregulated in HIST1H2BK-overexpressing PANC-1 cells compared with the control group, while no significant changes in other genes were detected (Fig. 5E). G9a participates in diverse biological processes, including DNA replication and DNA damage and repair, and catalyzes methylation processes such as methylation of p53, DNMT1, HDAC1 and KLF12 21-24. Additionally, G9a has been shown to be overexpressed in multiple human cancers and involved in tumor development 25-28, including in PDAC 29, 30. In conclusion, these results suggest that the oncogenic function of HIST1H2BK in PDAC might be related to its modulating effect on G9a.

### FAM110A is an essential target of TSPAN1 in PDAC

**Figure 6:**
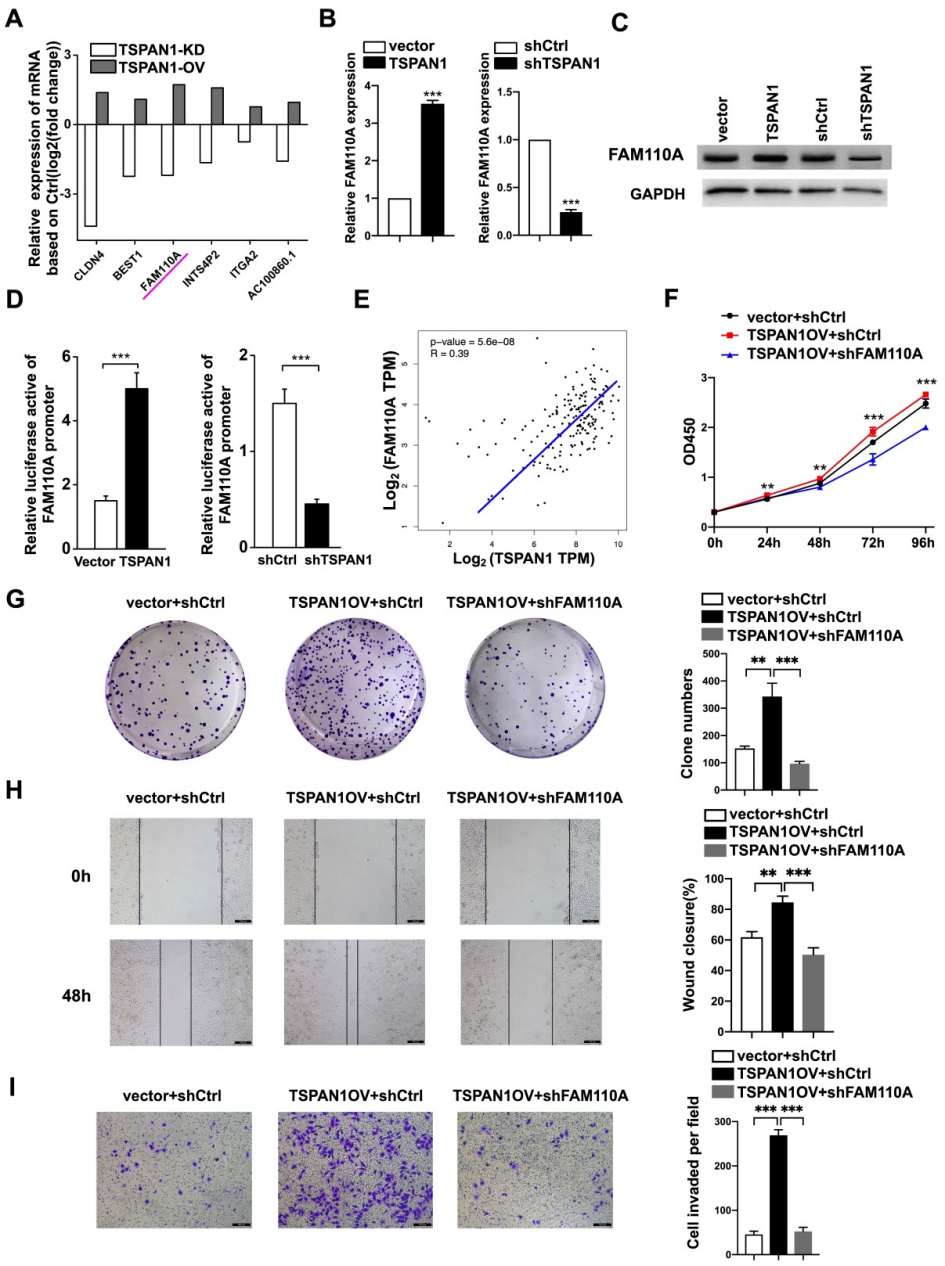
** FAM110A is responsible for TSPAN1-induced pancreatic cancer progression. A,** The relative mRNA levels of DEGs in PANC-1 cells after TSPAN1 overexpression or knockdown determined by RNA-seq compared with control cells. **B** and **C,** qPCR analysis (**B**) and Western blotting (**C**) of FAM110A in PANC-1 cells expressing vector, exogenous TSPAN1, shCtrl and shTSPAN1. **D,** A luciferase reporter assay evaluated the effect of TSPAN1 overexpression and knockdown on the transcriptional activity of FAM110A in PANC-1 cells. Firefly luciferase activity was normalized to Renilla luciferase activity and expressed as the mean ± SD. **E,** The correlation between TSPAN1 and FAM110A in PDAC based on the TCGA dataset. **F** and **G,** Cell proliferation (**F**) and colony-forming (**G**) capacity of PANC-1 cells with or without FAM110A knockdown in the presence of TSPAN1 overexpression. **H** and** I,** TSPAN1-overexpressing PANC-1 cells with or without FAM110A knockdown were analyzed in wound healing assays (**H**) and Transwell assays with Matrigel (**I**). Data are shown as the mean ± SD. n = 3 *P < 0.05, **P < 0.01 and *** P < 0.001 were considered significant.

TSPAN1 is a transmembrane protein that has been reported to be elevated in many human cancers and to be involved in tumor progression 8-12. According to our previous RNA-Seq with TSPAN1-overexpressing, shTSPAN1 and their corresponding control PANC-1 cells. We identified 6 genes positively regulated by TSPAN1 (fold change ≥2, P < 0.05), in which FAM110A presented identical trends as TSPAN1 in PANC-1 cells (Fig. 6A). Thus, TSPAN1 displayed the potent regulatory effect on FAM110A. In this study, we verified that TSPAN1 overexpression upregulated FAM110A expression, and shTSPAN1 decreased the expression of FAM110A at the mRNA and protein levels (Fig. 6B, C). TSPAN1 displayed a transcriptional regulatory effect on FAM110A based on a luciferase promoter-reporter assay. TSPAN1 overexpression increased the activity of the FAM110A promoter and vice versa (Fig. 6D). Our results are consistent with the correlation analysis based on the TCGA database, which exhibited a significant positive correlation between TSPAN1 and FAM110A mRNA levels in PDAC tissues (Fig. 6E). In our previous study, TSPAN1 was shown to be highly expressed in PDAC tissues and cells compared with noncancerous tissues and cells 8. Therefore, increased expression of the positive upstream regulator TSPAN1 may be one of the key reasons for FAM110A overexpression in PDAC.

To further determine the functional association with TSPAN1, we stably knocked down FAM110A in TSPAN1-overexpressing (OV) PANC-1 cells (PANC-1-TSPAN1OV-sh FAM110A). The PANC-1-TSPAN1OV-sh FAM110A cells showed a significantly slower proliferation rate (Fig. 6F, G) and attenuated migration and invasive abilities (Fig. 6H, I) compared with the PANC-1-TSPAN1OV-shCtrl cells. The tumor-promoting capacity of TSPAN1 was markedly abolished by FAM110A knockdown. In summary, these results indicate that the increased expression and oncogenic function of FAM110A in PDAC are closely related to its upstream regulator TSPAN1, which also plays a carcinogenic role in PDAC.

## Discussion

FAM110A belongs to the FAM110 family, which mainly functions in biological processes associated with the cell cycle, such as cell proliferation and differentiation. However, the role of FAM110A in cancer is still uncover. Our study demonstrated for the first time that FAM110A functions as an oncogene, promoting proliferation and metastasis in PDAC. The expression of FAM110A may be driven by TSPAN1, while the oncogenic function of FAM110A may be involved in the regulation of the histone H2B subtype HIST1H2BK. We also demonstrated for the first time the oncogenic role of HIST1H2BK in PDAC, which might be associated with its regulatory effect on G9a. This leads us to propose a new oncogenic TSPAN1/FAM110A/HIST1H2BK/G9a axis in PDAC that is involved in the modulation of tumor progression and represents a novel therapeutic approach for pancreatic cancer treatment.

The centrosome is the major microtubule (MT) organizing center. Centrosomes guide the assembly of interphase MTs associated with migration, morphogenesis and cellular signaling. Centrosome abnormalities can lead to a variety of diseases, including cancer 31. Cellular localization analysis showed that FAM110 proteins localized to centrosomes and accumulated at the microtubule organization center and spindle poles in interphase and mitosis 3. However, limited biological functions have been assigned to these proteins. This study proposes several important discoveries about the oncogenic functions and mechanisms of FAM110A in PDAC. We confirmed the abnormally elevated level of FAM110A in PDAC cells. We verified that the overexpression of FAM110A promotes PDAC proliferation, migration, invasion and tumorigenesis. This suggests that FAM110A plays an important role in tumor growth and may serve as a prognostic marker and therapeutic target for pancreatic cancer. It would be interesting to evaluate the role of FAM110A in other cancers in the future. In addition, we identified HIST1H2BK as the downstream target of FAM110A by RNA-seq. The luciferase promoter reporter assay showed that HIST1H2BK was transcriptionally regulated by FAM110A. Furthermore, we demonstrated that HIST1H2BK acts as an oncogene in PDAC, while the promotion effect caused by FAM110A overexpression could be reversed by HIST1H2BK knockdown. Therefore, we suggest that the oncogenic function of FAM110A in PDAC is closely related to its regulatory effect on HIST1H2BK.

Several modifications of histones have been well characterized, including methylation, acetylation, phosphorylation, adenylation, ubiquitination and ADP ribosylation. Aberrant histone modifications occur frequently in cancer and contribute to tumor initiation and progression 32. Mutations in the histones themselves have also recently been linked to cancers; namely, mutations in histone H2B at amino acid 76 (H2B-E76K) occur particularly in bladder, head and neck cancers 13. Moreover, some histone proteins are not widely distributed but are distributed in specific regions, such as HIST1H2BK, which is only found in histone microclusters on chromosome 6 p21.33. The reason for this regional distribution of such histones and its effect on gene expression in this region are not clear. Our study showed that overexpression of HIST1H2BK upregulated G9a, located in the HIST1H2BK distribution region, while other genes in the region showed no significant changes. However, the regulatory mechanism of HIST1H2BK and its other biological functions still need to be further explored.

TSPAN1 is the upstream regulator of FAM110A, and TSPAN1 transcriptionally regulates FAM110A. TSPAN1 overexpression significantly increased the levels of FAM110A mRNA and protein in PDAC cells. Moreover, we demonstrated that the oncogenic function of TSPAN1 in PDAC could be partially abolished by FAM110A knockdown. These results suggest that upregulated TSPAN1 may help drive PDAC by triggering FAM110A overexpression. It will be interesting to investigate the mechanism of how TSPAN1 regulates FAM110A expression in future studies. In our previous study, TSPAN1 was shown to regulate target genes in an epigenetic manner by modulating methyltransferase and demethylase levels 8. Whether TSPAN1 regulates FAM110A expression in the same way could be explored.

## Conclusions

In conclusion, this is the first demonstration of the significance of FAM110A in pancreatic cancer. Our work elucidated the critical role of FAM110A in tumorigenesis and prognosis, whereby FAM110A reinforced cell proliferation, migration, invasion and tumorigenesis both *in vitro* and *in vivo*. TSPAN1 is an upstream regulator of FAM110A, and FAM110A regulates the downstream transcriptional activity of HIST1H2BK, which could abolish the functions of FAM110A in PDAC. We have also shown, for the first time, that HIST1H2BK functions as an oncogene in PDAC, which might be associated with its modulatory effect on G9a. Collectively, the newly identified TSPAN1/FAM110A/HIST1H2BK/G9a axis promotes PDAC progression and represents a novel therapeutic strategy against pancreatic cancer.

## Supplementary Material

Supplementary figures and tables.
