# Supplementary material for: TSPAN1-elevated FAM110A promotes pancreatic cancer progression by transcriptionally regulating HIST1H2BK
Source: J Cancer. 2022 Jan 1;13(3):906–17. doi: 10.7150/jca.66404 (PMC8824879; doi:10.7150/jca.66404)
Supplement: Supplementary file 1 — Supplementary figures and tables. [file jcav13p0906s1.pdf]

Supplementary Figure S1

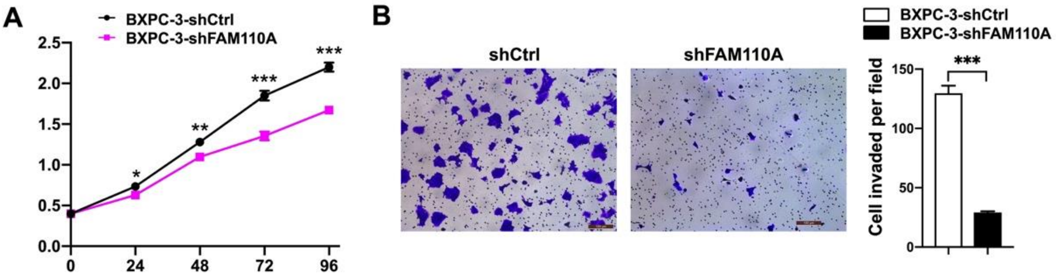

Figure S1. FAM110A enhances the proliferation and invasion of pancreatic cancer.

A, Proliferation of BXPC-3 cells expressing shFAM110A and shCtrl. **B,** Transwell assays were used to evaluate the invasive potential of FAMM110A knockdown and control cells (scale bar: 100 μm). shFAM110A inhibited the proliferation and invasion of BXPC-3 cells. Data are shown as the mean ± SD. n = 3 *P < 0.05, **P < 0.01 and *** P < 0.001 were considered significant.

Supplementary Figure S2

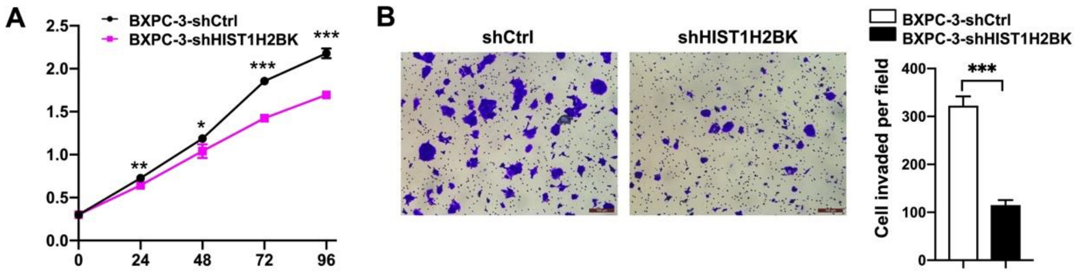

Figure S2. HIST1H2BK enhances the proliferation and invasion of pancreatic cancer.

A, Proliferation of BXPC-3 cells expressing shHIST1H2BK and shCtrl. **B,** Transwell assays were used to evaluate the invasive potential of HIST1H2BK knockdown and control cells (scale bar: 100 μm). shHIST1H2BK inhibited the proliferation and invasion of BXPC-3 cells. Data are shown as the mean ± SD. n = 3 *P < 0.05, **P < 0.01 and *** P < 0.001 were considered significant.

Supplementary Figure S3

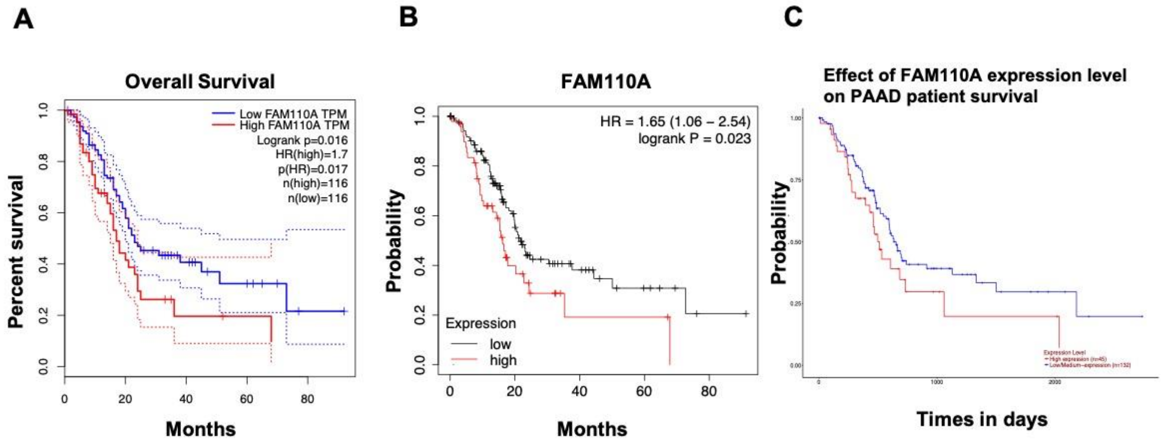

Figure S3. Bioinformatics analysis indicated that FAM110A was correlated with poor survival.

A GEPIA, **B** Kaplan–Meier Plotter and **C** Ualcan databases were used for survival analysis to evaluate the influence of FAM110A on the overall survival of PC patients.

Supplementary Figure S4

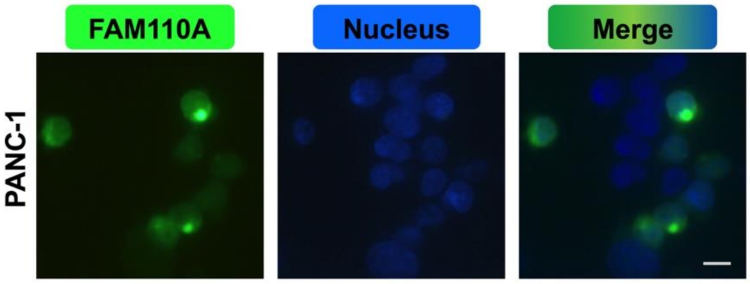

Figure S4. Subcellular localization of FAM110A in PANC-1 cells.

Immunofluorescent images of FAM110A (green) in PANC-1 cells. Nuclei stained with DAPI in blue. Scale bar = 10 µm.

Supplementary Table S1. Primer sequences for quantitative PCR

Target Gene	Primers (5'-3')
β-Actin	F: CATGTACGTTGCTATCCAGGC<br>R: CTCCTTAATGTCACGCACGAT
HIST1H2BK	F: CGACATCTTCGAACGCATCG<br>R: GTGTACTTGGTGACGGCCTTG
FAM110A	F: GTCCCTGGCTACCTGCTAC<br>R: CTGTCACACAAGTCGATGAGG
EHMT2	F: TCCAATGACACATCTTCGCTG<br>R: CTGATGCGGTCAATCTTGGG
POU5F1	F: GGGAGATTGATAACTGGTGTGTT<br>R: GTGTATATCCCAGGGTGATCCTC
HSPA1L	F: TTACCGTGCCAGCCTATTTCA<br>R: AGCACATTAAGTCCAGCAATCA
TNF-F	F: GAGGCCAAGCCCTGGTATG<br>R: CGGGCCGATTGATCTCAGC
DDR1	F: CCGACTGGTTCGCTTCTACC<br>R: CGGTGTAAGACAGGAGTCCATC
TNXB	F: GCCCTGCTCACTTGGACTG<br>R: GGAGCCGTGCATTGTAGGAG
STK19	F: GACCTTTGGAGTTAAGAGGCG<br>R: CTGGACGATTCTGATCTCCCC
CYP21A2	F: CAAGCTGGTGTCTAGGAACTACC<br>R: TCTCATGCGCTCACAGAACTC
SLC44A4	F: CAAGGCTGTGGGACAGATGAT<br>R: CCCAGTAGGCAATGCAGATGA
CLIC1	F: CTGGGCTGGACATATTTGCCA<br>R: GCTCGTTGCCATCCAAAAACT
TCF19	F: TTACCATCCCACGGTCTAGGG<br>R: GCTGCCTATGGAGTTTAGGATCA

F, Forward; R, Reverse

Supplementary Table S2. Characteristics of antibodies used in the study

Antigen or description	Application	Origin	Dilution
Primary antibodies			
FAM110A	WB	orb166688, Biorbyt, UK	1:1000
HIST1H2BK	WB	orb548476, Biorbyt, UK	1:5000
P53	WB	orb99409, Biorbyt, UK	1:1000
CDKN1A	WB	orb48324, Biorbyt, UK	1:1000
PCNA	WB	orb48485, Biorbyt, UK	1:1000
BAX	WB	orb31066, Biorbyt, UK	1:1000
BCL2	WB	orb99416, Biorbyt, UK	1:1000
Beta-Actin	WB	orb378579, Biorbyt, UK	1:5000
GAPDH	WB	Orb555879, Biorbyt, UK	1:5000
Secondary antibodies			
Goat Anti-Rabbit IgG antibody (HRP)	WB	orb43514, Biorbyt, UK	1:5000
Goat Anti-Mouse IgG (H+L) antibody (HRP)	WB	orb506151, Biorbyt, UK	1:5000

HRP, horseradish peroxidase; WB, Western blotting

Supplementary Table S3.

Surgical specimens of pancreatic cancer and peritumor samples

Sample No.	Age	Sex	Pathology diagnosis	Grade	Tumor diameter
01	62	Male	pancreatic ductal adenocarcinoma	II	17
02	50	Male	pancreatic ductal adenocarcinoma	II	24
03	57	Female	pancreatic ductal adenocarcinoma	II	18
04	51	Male	pancreatic ductal adenocarcinoma	I	12
05	42	Female	pancreatic ductal adenocarcinoma	II	23
06	52	Male	pancreatic ductal adenocarcinoma	I	10
07	61	Female	pancreatic ductal adenocarcinoma	I	14
08	60	Male	pancreatic ductal adenocarcinoma	I	15
09	56	Male	pancreatic ductal adenocarcinoma	II	21
10	58	Male	pancreatic ductal adenocarcinoma	I	16
11	39	Female	pancreatic ductal adenocarcinoma	II	20
12	65	Male	pancreatic ductal adenocarcinoma	I	13
